# Molecular Profiling of Thyroid Nodules: Current Role for the Afirma Gene Expression Classifier on Clinical Decision Making

**DOI:** 10.4274/2017.26.suppl.05

**Published:** 2017-01-09

**Authors:** Richard T. Kloos

**Affiliations:** 1 Veracyte Inc., Department of Medical Affairs, Senior Medical Director-Endocrinology, California, USA

**Keywords:** Biopsy, fine-needle aspirate, Gene expression, Genomics, molecular diagnostic techniques, Thyroid nodule

## Abstract

Thyroid fine-needle aspiration biopsy results are cytologically indeterminate in 15-30% of cases. When these nodules undergo diagnostic surgery, approximately three-quarters are histologically benign. These unnecessary surgeries diminish quality of life, generate complications, and increase healthcare costs. The Afirma gene expression classifier (GEC) is validated to pre-operatively identify cytologically indeterminate nodules likely to be truly benign so that surgery can be avoided. Its performance is supported by robust multicenter prospective and blinded clinical validation studies, and supported by extensive independent clinical utility publications which show a marked reduction in surgery among patients with benign Afirma GEC results. To rule-out cancer and avoid unnecessary diagnostic surgery, Afirma’s quality and depth of validation stand alone. The accuracy of a benign result is the negative predictive value (NPV). Afirma achieves an NPV ≥94% among cytologically indeterminate nodules (Bethesda III or IV). Thirteen clinical utility studies describing 1468 GEC benign patients demonstrate that few Afirma GEC benign nodules undergo surgery, including after 3 years of follow-up. With a specificity of 52%, over half of the truly benign nodules with indeterminate cytology receive a benign GEC result. High test sensitivity is critical to safely rule out cancer. The Afirma GEC’s 90% sensitivity means that regardless of the pre-test risk of malignancy, 90% of all malignant nodules are GEC suspicious. The Afirma GEC has transformed patient care. Where the majority of cytologically indeterminate patients were once operated to determine if the nodule was benign or malignant, now nearly half of these surgeries can be avoided.

## Abstract (tr)

Belirsiz sitoloji tiroid ince iğne biyopsi örneklerinin %15-30’unda bulunur. Bu nodüller tanı amaçlı ameliyat edildiğinde yaklaşık dörtte üçünün histolojik olarak benign olduğu saptanmıştır. Gereksiz ameliyatlar yaşam kalitesini azaltır, komplikasyonlara neden olur ve sağlık giderlerini artırır. Afirma gen ekspresyonu sınıflandırıcı [Gene Expression Classifier (GEC)] operasyon öncesi benign olma olasılığı olan belirsiz sitolojili nodülleri tespit ederek cerrahi girişim gerekliliğini önleyebilir. Testin etkinliği güvenilir çok merkezli prospektif kör klinik doğrulama çalışmalarıyla, benign Afirma GEC sonuçları ile bu hasta grubunda cerrahinin belirgin bir azalma gösterdiği ise geniş, bağımsız klinik yarar yayınları ile desteklenmiştir. Kanseri ekarte etmek ve gereksiz tanısal cerrahiyi önlemek amacıyla Afirma, kalitesi ve doğrulama oranı ile alanında tektir. Benign bir sonucun doğruluğu testin negatif prediktif değerini (NPD) gösterir. Afirma’nın NPD’yi belirsiz sitolojili nodüller için (Bethesda III veya IV) ≥%94’e ulaşmaktadır. GEC benign 1,468 hastayı içeren on üç klinik yarar çalışması, sadece birkaç Afirma GEC benign nodülde ameliyat ihtiyacı olduğunu 3 yıllık takip süresi ile göstermektedir. Testin %52 özgüllüğü ile, gerçekten benign olan belirsiz sitolojili nodüllerin yarısından fazlasında benign GEC sonucu alınmaktadır. Kanseri güvenle ekarte etmek için yüksek test duyarlılığı kritik öneme sahiptir. Afirma GEC’nin %90 duyarlılığı, test incesi malignite riski ne olursa olsun, tüm malign nodüllerin %90’ının GEC şüpheli olduğu anlamına gelir. Afirma GEC hasta yaklaşımını değiştirmiştir. Belirsiz sitolojili hastaların çoğunluğu nodülün iyi veya kötü huylu olup olmadığını belirlemek için ameliyat edilirken, günümüzde bu ameliyatların neredeyse yarısı önlenebilir.

## INTRODUCTION

**Figure 1:**
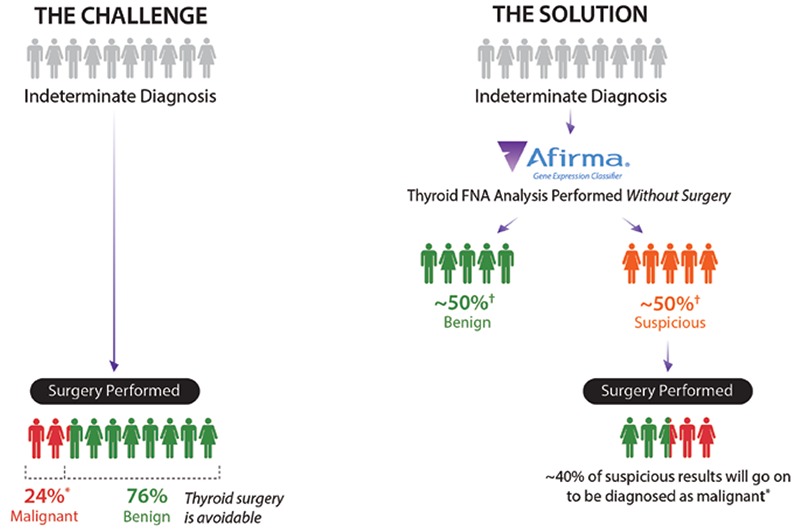
Afirma gene expression classifier reduces unnecessary thyroid surgeries compared to management without gene expression classifier testing *Alexander et al. (4), †see Figure 3

Prior to the adoption of thyroid nodule fine-needle aspiration biopsy (FNAB), thyroid nodules were regularly referred for diagnostic surgery because of their 5-15% risk of malignancy (ROM) (1). FNAB decreased diagnostic thyroidectomies by one-half as most FNAB results are cytologically benign and surgery is typically avoided (2). Still, 15-30% of thyroid FNABs are cytologically indeterminate, i.e. not clearly benign nor malignant (1,3). When cytologically indeterminate thyroid nodules undergo diagnostic surgery, approximately three-quarters prove to be benign on surgical histopathology (Figure 1) (4,5,6). The care of such patients is being dramatically altered by a new diagnostic strategy that pre-operatively identifies many of these benign nodules with indeterminate cytopathology [Bethesda categories III and IV (7)] as having a low risk of cancer so that diagnostic surgery can be avoided, along with its costs, complications, and inconveniences. Complications from thyroid surgery include, but are not limited to, hypothyroidism, voice changes, vocal cord dysfunction, hypocalcemia (temporary and permanent), tracheostomy, hematoma, infection, hospital readmission, and death. Complications are highest in patients older than 65 years of age, and when the procedure is performed outside of high-volume thyroidectomy hospitals (8). Among cytologically indeterminate nodules, patient clinical factors, ultrasound characteristics (9), additional cytological subcategorization or second opinion, and repeat FNAB have been unable to reliably identify a significant fraction of benign nodules to safely avoid surgery. For example, among Bethesda III nodules, those with any ultrasound predictive feature (solid, hypoechoic, microcalcifications, increased vascularization, or irregular margin) were found to have at least a 12% ROM, which increased further when additional features were present (10). Current excitement has focused on molecular genomics approaches. To date, only the Afirma gene expression classifier (GEC) (Veracyte Inc., South San Francisco, California) is supported by prospective, multicenter, and blinded validation studies to reclassify nodules as benign, and has been shown in multiple clinical utility studies to reduce avoidable diagnostic surgeries based on the test result.

## TESTS TO RULE-IN AND RULE-OUT CANCER

A test with a high sensitivity and high negative predictive value (NPV) is able to rule-out cancer (11,12). Test sensitivity measures the fraction of cancers that the test identifies as “positive” (e.g. Afirma GEC suspicious). Afirma GEC test sensitivity among indeterminate nodules is 90% (4). Test NPV measures the fraction of “negative” calls by the test (e.g. Afirma GEC benign) that are correct. Afirma GEC test NPV is 94-95% amongst Bethesda III and IV nodules at a cancer prevalence of 24-25% (4). While not mutually exclusive, a test with a high specificity and high positive predictive value (PPV) is able to rule-in cancer. Test specificity measures the fraction of benign nodules that are called benign by the test. Afirma GEC test specificity is 52% (4), suggesting that just over half of the benign nodules are called GEC benign. Test PPV measures the fraction of “positive” calls by the test (e.g. Afirma GEC suspicious) that are correct. Afirma GEC test PPV is 37-38% amongst Bethesda III and IV nodules (4). Thus, the strength of the Afirma GEC is its ability to rule-out cancer (NPV), more than its ability to rule-in cancer (PPV). A rule-in test is of value when it changes clinical care, such as altering the extent of thyroid surgery from a lobectomy to a total thyroidectomy. However, the necessity of total thyroidectomy for patients with thyroid cancer less than 4 cm, without gross extra-thyroidal extension, distant metastases, or clinically apparent metastases to the lymph nodes has not been established and current guidelines do not mandate total thyroidectomy in the absence of these features (13). Thus, the utility of rule-in tests is currently questioned as patient benefit has not been established. Given the modest specificity and PPV of Afirma, it is not considered a rule-in test. While an Afirma GEC suspicious result raises the risk of cancer from 24-25% to 37-38%, it should be clear that the strength of the test is that it identifies just over one-half of all benign nodules with Bethesda III or IV cytology as genomically benign, and 90% of all cancers as genomically suspicious regardless of the cancer prevalence (Figure 1). Thus, when applied to the typical cytologically indeterminate nodule with ROM of 25% or less, the expected accuracy of a benign result (NPV) is 94% or greater. As a result, most Afirma GEC benign nodules are candidates for clinical observation in lieu of diagnostic surgery. Additional “cassettes” are tested with every Afirma GEC to identify rare neoplasms that are often difficult to accurately diagnose with cytology such as medullary thyroid cancer (MTC), parathyroid neoplasms, and metastases to the thyroid from malignant melanoma, breast, and renal cell carcinomas. Failing to trigger one of these cassettes, the GEC evaluates the expression of 142 genes that are used in a proprietary mathematical algorithm to classify indeterminate thyroid nodule samples as either GEC benign or GEC suspicious.

## RATIONALE FOR THE MEASUREMENT OF MESSENGER RIBONUCLEIC ACID EXPRESSION

The Afirma GEC is based on the measurement of messenger ribonucleic acid (mRNA) expression. There are several diagnostic advantages to using RNA instead of other approaches such as DNA mutations or microRNA expression. Unlike cancers whose cytology is Bethesda V or VI, cancers that are cytologically indeterminate (Bethesda III and IV) typically lack the most common genomic abnormality of differentiated thyroid cancer: BRAFV600E mutation. In its absence, the most common classic mutation amongst cytologically indeterminate cancers are RAS mutations, but these are found in the minority and are also found in benign nodules. As benign nodules outnumber malignant nodules 4:1 among nodules with indeterminate cytology, the PPV of RAS mutations is poor in a number of studies (14,15,16,17,18,19,20,21). Herein lies the challenge of mutational approaches for cytologically indeterminate nodules: many malignancies lack the known genomic abnormalities (22,23), and when present, most genomic abnormalities are not specific for cancer (22,23).

While there are only approximately 23,000 known protein-coding DNA genes (24), each of these may be transcribed into multiple alternatively-spliced variants, with more than 240.000 known mRNA isoforms. Disease-causing DNA alterations generally exert their effects, at least partially, on the transcriptome. Similarly, microRNAs impart their effects by altering transcription. Therefore, mRNA expression provides a cumulative measurement of various known (and unknown) upstream effects. Additionally, gene expression may be impacted by lifestyle and environmental factors so that mRNA expression reflects additional significant information not discernible from DNA or microRNA analysis alone.

Gene expression classifiers quantitatively evaluate the relative expression levels of multiple genes that comprise the genomic signature of the interrogated tissue. In the development of Afirma GEC, instead of discriminately relying on genes previously identified in the literature, analysis of the whole genome (transcriptome) was used to identify candidate genes, and support vector machine learning methods were used to develop the classifier algorithm (4,25). The genes utilized in the cassettes and main Afirma GEC classifier have been published (4). This powerful methodology more fully utilizes the genomic information of the biological sample than is used by target next generation sequencing approaches.

## CLINICAL VALIDATION

Physicians find risk of cancer associated with a cytological benign FNAB diagnosis to be low enough to defer surgery in the vast majority of such patients. A 6-8% risk of cancer among operated cytology benign nodules has been described (4,5,26,27,28,29,30). Thus, a test that could reliably identify cytologically indeterminate nodules with a similar or lower risk of cancer (e.g. NPV ≥94%) could allow these nodules to also be considered for clinical observation instead of diagnostic surgery. Clinical validation of the Afirma GEC was initially performed on a small independent sample set of thyroid nodule FNABs within a prospective multicenter, double blind study design (25). The Afirma GEC achieved high sensitivity and NPV, including among cytologically indeterminate nodules. After further optimization, the GEC was validated in a second larger independent sample set in a prospective multicenter validation study. The second study included the largest ever prospectively collected set of thyroid FNAB biopsies from 3,789 unique patients, with a final validation set of 265 cytologically indeterminate nodules. Based on the 24% prevalence of malignancy in cytologically indeterminate samples (Bethesda III+IV), a 95% NPV for the Afirma GEC was achieved (4).

The unique and often overlooked strength of this prospective, multicenter, and blinded validation design is that it supports generalizability of the results. Prospective and multicenter study designs reduce selection bias and better represent what is likely to occur in real-world practice. The 3.789 patients were prospectively consented and enrolled in the trial before undergoing FNAB at 49 study sites across the country, including academic and community practices, which provides confidence in the external validity of the findings. Strong internal validity was demonstrated when no differences were found between the final validation cohort of 265 patients compared to the full prospective and consecutive total enrollment cohort in patient age, gender, cancer risk factors, or nodule size. As investigators were blinded to the Afirma GEC result, the test result did not influence which patients underwent surgery. These important study design elements (prospective, blinded, and multicenter) support the internal and external validity of the study, and provide confidence in the broader generalizability of the study findings to a physician’s own clinical practice (13). In contrast, significant biases can be introduced when the study cohort does not mimic the intended use cohort. For example, profound bias can occur in unblinded studies where the test result influences inclusion or exclusion from the “validation” cohort (13). The Afirma GEC is the only test for cytologically indeterminate nodules demonstrated to have an accurate enough benign result (e.g. NPV ≥94%) proven in a rigorous and published prospective, blinded, and multicenter validation study to allow physicians to strongly consider clinical observation instead of surgical resection for Bethesda III and IV nodules (4).

Overall, the ROM for a thyroid nodule with Bethesda categories III and IV indeterminate cytology with an Afirma GEC benign classifier result is about 5% (1-NPV). This risk is comparable to the 6-8% cancer risk for an operated thyroid nodule with a benign cytology diagnosis (4,5,26,27,28,29,30). This demonstrates that cytologically indeterminate nodules (Bethesda categories III and IV) with an Afirma GEC benign diagnosis can be managed as would a cytologically benign nodule (4,31), as suggested by the National Comprehensive Cancer Network (NCCN) Thyroid Carcinoma Guideline (32). In contrast, others have attempted to create rule-out tests using the most common DNA point mutations, fusions, or proprietary microRNA signatures where the false negative rate may be unacceptable for routine clinical use. Asuragen reported in its prospective, multicenter, and blinded 7-gene mutation panel study that it missed as many as 53% of malignant Bethesda III and IV nodules (33), a rate significantly higher than had been seen in an earlier unblinded single center study (34). Interpace reported that its 8-gene mutation panel (ThyGenX) missed 40% of malignant nodules (35), while independent studies (22,23) have not confirmed claims of improved sensitivity and specificity with even larger mutation/fusion panels (36,37). Interpace has combined ThyGenX with a 10 microRNA classifier and in a second study reported that it missed 20% of malignancies (35). Similarly, Rosetta Genomics reported high sensitivity of its microRNA classifier when 20% of samples (1 in five cases) were excluded based on lack of histological agreement amongst 3 pathologists. In practice, physicians can’t know which patients to exclude pre-operatively, so test performance is more accurately reflected amongst the entire cohort where nearly 1 in 6 cancers were missed (38).

## CLINICAL PRACTICE EXPERIENCES AND CLINICAL UTILITY

**Figure 2:**
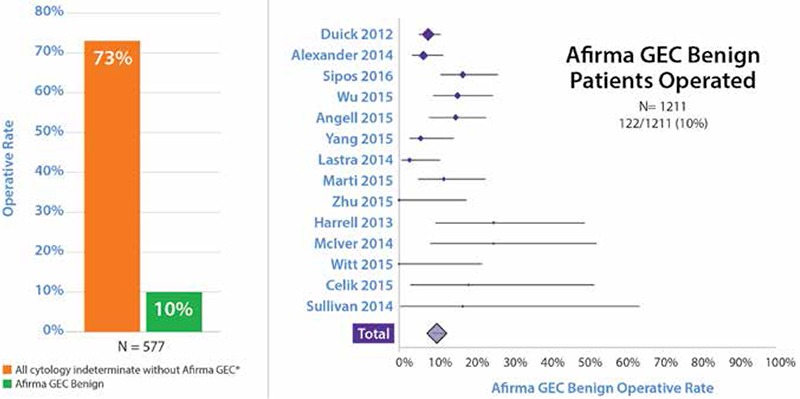
Real-world clinical utility *Cibas et al. (52). Afirma gene expression classifier benign operative rate references (9,31,40,41,42,43,44,45,46,47,48,49,50,51,), GEC: Gene expression classifier

While clinical validation demonstrates the test’s ability to accurately predict the diagnosis, clinical utility measures the test’s impact on real-world patient management decisions and impact on net health outcomes (39). Fourteen publications now describe the clinical experience with the Afirma GEC in routine clinical practice (9,31,40,41,42,43,44,45,46,47,48,49,50,51). Among the Afirma GEC benign patients, only 122 of the 1211 patients (10%) were operated, demonstrating a dramatic reduction in surgery compared to the 73% historical rate of surgery (52) when Afirma was not used (Figure 2). Five of the Afirma GEC publications were multicenter (40,41,43,46,51), two had a minimum follow-up of 1 year (45,48), and one reported patients Afirma GEC tested at least 3 years prior to study enrollment (43). In that study, 17% of Afirma GEC benign patients underwent surgery and 88% of the surgeries occurred within 2 years of the biopsy. Yang et al. (50) reported that surgery was significantly reduced in both Bethesda III and IV categories when they globally compared patient management before and after implementation of Afirma GEC testing. Overall, the findings demonstrated a durable and dramatic reduction in diagnostic surgery.

**Figure 3:**
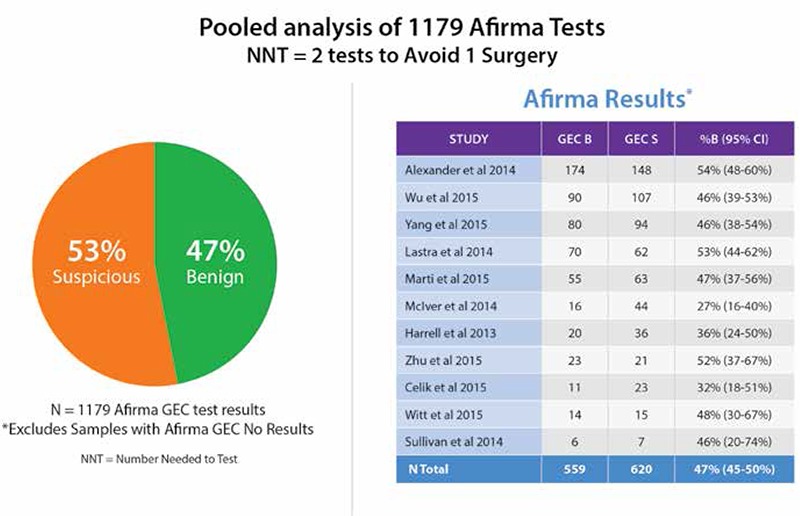
Eleven independent clinical utility studies. Afirma gene expression classifier result rate references (9,41,42,44,45,46,47,48,,49,50,51) GEC: Gene expression classifier

Two cohorts of 2667 (40) and 2040 GEC resulted tests (53) have reported 53% and 52% as GEC benign, respectively. Eleven independent publications report their frequencies of benign versus suspicious GEC results: 47% of 1179 tests amongst cytologically indeterminate nodules were GEC benign (Figure 3) (9,41,42,44,45,46,47,48,49,50,51). Defining the number needed to test (NNT) as the number of tests needed to be performed to change the clinical outcome of one patient (NNT=1/(%GEC benign), and rounded to the nearest whole person), then the NNT of these series is 2. Consequently, one patient potentially avoids surgery for every two patients tested (Figure 1).

As noted above, clinical experience/clinical utility studies serve an important role in the chain of evidence regarding the effectiveness and value of a test. These allow medical centers and community practices to describe the impact the GEC has had in their management setting. As more long-term follow-up data becomes available, these important studies will allow researchers to model the overall GEC impact on reducing unnecessary surgeries nationally.

It is important to note, however that most GEC benign patients in the clinical series reported to date did not undergo surgery, consistent with the purpose of the test (9,31,41,42,43,44,45,46,47,48,49,50,51). Thus, such clinical experience studies cannot serve as proper clinical validation studies, and clinicians should be wary when attempts are made from such studies to measure or question test performance. Operated GEC benign patients alone in a clinical experience series are insufficient to evaluate test performance, and these patients often differ from the broader group of GEC benign patients, and are more likely to include those nodules at greater risk of cancer. Therefore, findings among these patients are unlikely to be generalizable to the majority of the GEC benign case. Any attempt to measure test performance such as sensitivity and NPV requires operating on all tested patients in a contiguous intended use cohort with centralized blinded histology (e.g. clinical validation).

**Figure 4:**
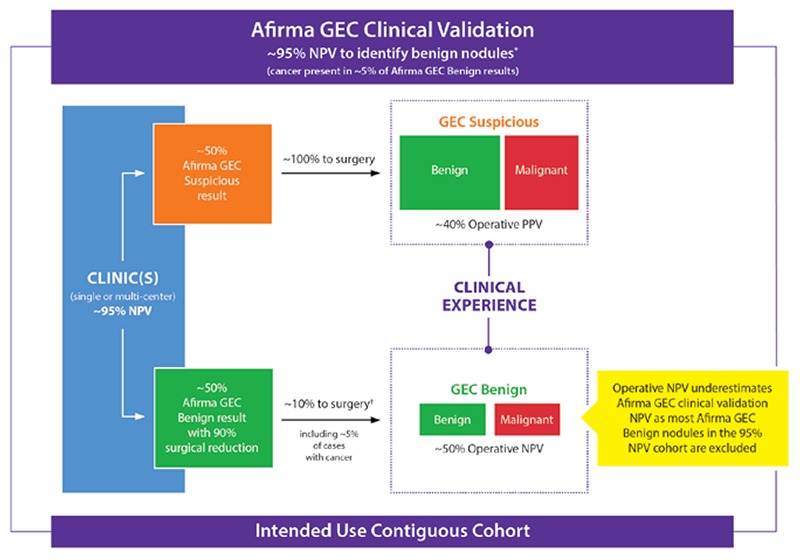
Afirma gene expression classifier clinical validation negative predictive value versus hypothetical “operative negative predictive value”. These phenomena co-exist, rather contradict each other. Outside of a properly designed clinical validation study (Figure 5), the “operative negative predictive value” from clinical experience studies creates significant confusion while offering little clinical value *Alexander et al. (4), †Afirma gene expression classifier benign cases referred to surgery due to ultrasound features, ultrasound changes over time, nodule growth, etc., GEC: Gene expression classifier

**Figure 5:**
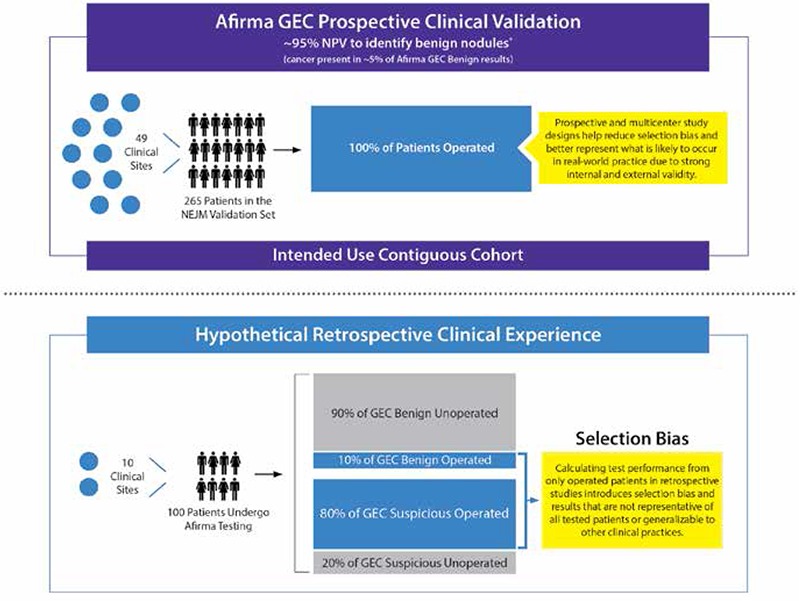
Clinical experience studies do not control for selection bias. Test performance is properly measured in clinical validation studies, while clinical utility is measured in clinical experience studies. *Alexander et al. (4) GEC: Gene expression classifier, NPV: Negative predictive value

Additionally, clinical experience series may differ from properly performed prospective validation studies as the former may not report on a consecutive cohort of tested patients from the catchment area, but rather report only on patients who come to their attention through a variety of referral patterns. Thus, the cohort described may not reflect how the test works in the intended use population. Figure 4 describes hypothetically how clinical experience studies that generate “operative NPV” results that may appear to conflict with the published 95% clinical validation NPV, but rather co-exists within the larger 95% NPV clinical validation experience. The operative NPV experience reflects the selection bias that occurs when higher ROM GEC benign patients are selected for surgery out of good clinical judgement while not operating on all continuous GEC benign patients from the entire referral base. While the operative NPV from clinical experience studies is easy to calculate, it generates great confusion for the usual reader while actually offering little clinical meaning when generated outside of a comprehensive clinical validation study. This discussion (and Figure 5) highlight the importance of study design, and the potential misinterpretations of data that can emerge from clinical experience studies.

**Figure 6:**
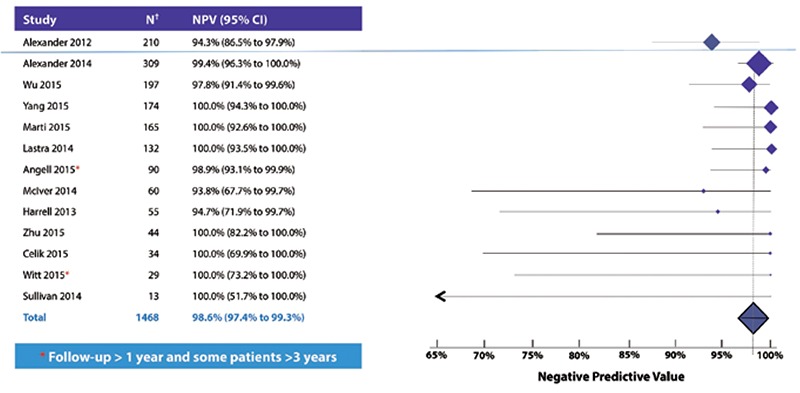
Consistently high estimated negative predictive value across 13 real-world studies. Negative predictive value calculated as true negatives (Afirma gene expression classifier benign and either unoperated or operated and histopathologically benign) divided by all gene expression classifier benign results †Includes Bethesda III (atypia/follicular lesion of undetermined significance) and IV (follicular/Hürthle cell neoplasm). Figure updated from Steward and Kloos (53). References (4,9,31,41,42,44,45,46,47,48,49,50,51), NPV: Negative predictive value, CI: Confidence interval

Another limitation of clinical experience studies is that when Afirma GEC suspicious nodules are unoperated then test sensitivity among the operated cases is likely to be reduced (Figure 5). More importantly, exclusion of unoperated GEC benign nodules excludes a large number of truly benign nodules, which dramatically reduces estimates of specificity and NPV (11,42,46,50,51,54). However, as most cytologically indeterminate nodules are histologically benign, and because two clinical validation studies demonstrated a high NPV for Afirma, performance can be estimated amongst the 1468 GEC tested cytologically indeterminate patients in the published literature by pooling them together and considering GEC benign patients with malignancy found at surgery (ten patients) as malignant (false negatives), and GEC benign patients that underwent surgery and were histologically benign as benign (true negatives), or were GEC benign and not operated (704 patients). Among these GEC tested patients across multiple clinical practices, the pooled accuracy of a GEC benign result (NPV) was >98% (95% confidence interval (CI) 97-99%) (Figure 6) (4,9,31,41,42,44,45,46,47,48,49,50,51). These findings across academic and community-based practices are consistent with each other and the clinical validation of Alexander et al. (4) revealed an NPV of 94%. Two of the studies report a median follow-up of 1 year, while some patients had been followed more than 3 years. While it is true that some of the unoperated GEC benign patients may eventually be found to harbor malignancy over time, the consistently high estimated NPV seems unlikely to decline significantly. These data demonstrate a very low prevalence of malignancy (1-NPV) in patients with cytologically indeterminate thyroid nodules that are Afirma GEC benign, and support clinical observation in lieu of diagnostic surgery for most GEC benign patients.

The accuracy of an Afirma GEC benign call (NPV) remains high amongst Hürthle cell cytology, although the rate of benign calls is lower. Hürthle cell cytology has been a challenge for molecular diagnostics. Performance can be increased by removing these samples from clinical testing (55), but this does not help the clinician who must manage these patients. There is an overlap in the molecular profiles of benign and malignant samples. To maintain the accuracy of a benign call, the GEC can only call about half of all Bethesda III and IV samples GEC benign (Figure 3). The overlap is even greater among Hürthle cell samples. Thus, to maintain the accuracy of a benign call, the GEC calls fewer samples as GEC benign, and more samples suspicious. Among 5 cohorts of Hürthle cell samples totaling 378 nodules with an Afirma GEC benign or suspicious result, 147 (39%) were called GEC benign (42,47,53,56). Thus, three patients must be tested to avoid 1 surgery. Some observers have lamented that most Afirma results are suspicious in these cases while the prevalence of malignancy at surgery remains low within this group. However, there is no other validated method to determine which of these cases can safely avoid surgery. Brauner et al. (56) reported in a multicenter study of Massachusetts General Hospital, Brigham and Women’s Hospital, and Beth Israel Deaconess Medical Center that only 3 of 26 Afirma GEC benign nodules underwent surgery (12%), and all were benign at surgical pathology, consistent with a high NPV. Including all Afirma benign and suspicious results, use of Afirma reduced the overall operative rate from 80-81% among two control groups, to 65% when the Afirma GEC was used. To date only one false negative (malignant) Hurthle cell nodule has been called benign by the Afirma GEC in a published study (4). This high degree of accuracy among Afirma GEC benign results is remarkable given the typical high degree of disagreement at surgical pathology over a benign or malignant diagnosis (52).

## IMPLEMENTATION IN ROUTINE CLINICAL PRACTICE

Physicians collect two extra FNAB passes for potential molecular testing with the Afirma GEC on every FNAB they perform, or have on-site rapid cytological assessment so that the GEC can be collected on every patient with indeterminate cytology during one patient visit (Figure 1). This patient-centric approach avoids the inconvenience, delayed diagnosis, and costs associated with repeating the FNAB should the first FNAB cytology results be indeterminate. The passed collected for Afirma are immediately placed in the Veracyte-provided RNA protective solution tube for storage and chilled box shipping (<25 °C).

It is well known that cytologically indeterminate nodules may not be categorized as indeterminate if they undergo a repeat FNAB (57). While the hope of repeating the FNAB is to re-stratify cytologically indeterminate nodules as either cytologically benign or malignant, the ability of a cytology benign result on the second FNA to safely avoid surgery is unproven. Studies on this topic are imperfect as not all patients undergo surgery to establish histological truth, however, several studies indicate a ROM amongst nodules with a Bethesda III cytology followed by a benign cytology diagnosis that is between the risk of the two categories (44,57,58), with the highest being a 29% risk of cancer (57). Some evidence suggests that the same is true when one pathologist over-reads a cytologically indeterminate sample as cytologically benign (59). Indeed, investigators from Johns Hopkins University reported 7 operated patients with cytologically indeterminate FNAB findings and Afirma GEC suspicious results where their cytologists pre-operatively changed the cytology diagnosis to benign. Surgical pathology revealed malignancy in 29% of these cases (54). Thus, the risk of cancer among nodules with a benign cytology result after a repeat FNAB or after review by another cytopathologist, may exceed the ~5% or less ROM threshold of the NCCN to consider nodule observation (32,60). Similarly, the 2015 American Thyroid Association (ATA) guideline recognized these considerations against the role of repeat FNAB (13). Given the risk that a repeat FNAB may not eliminate the need for surgery, and the typical dislike of the FNAB procedure itself, some patients seek care elsewhere or elect diagnostic surgery rather than repeat FNAB. This seems like a lost opportunity as many of these patients may have benefitted from utilizing the Afirma GEC. For these reasons, it is strongly recommend that the GEC specimen be collected at the same time as the cytology sample during the first thyroid FNAB.

## CLINICAL DECISION MAKING

The PPV and NPV are determined by the pre-test ROM. To practice personalized medicine, it is important to consider the individual patient’s pre-test risk. The patient’s pre-test ROM includes their individual features (e.g. gender, history of childhood radiation treatment, ultrasound findings, serum TSH, etc.) and the interpreting cytologist’s thresholds to utilize cytology indeterminate categories. Ignoring this step of personalized care and assuming that every patient at a practice or institution has the same pre-test risk ignores important medical information.

The 2015 ATA guidelines allow for either hemithyroidectomy or near-total/total thyroidectomy for thyroid malignancy 1-4 cm in size without gross extra-thyroidal extension or clinical evidence of lymph node metastases (13). Thus, multiple factors must be taken into consideration when planning surgical intervention for cytologically indeterminate nodules, such as the risks and benefits, the presence of significant contralateral nodules, long-term follow-up, the role for completion thyroidectomy with or without radioactive iodine ablation if malignancy is found, and patient preferences.

The 2015 ATA guideline emphasizes ultrasound characteristics to predict the nodule’s ROM (13). Afirma is expected to identify 90% of cancers as GEC suspicious, and 52% of the benign nodules as GEC benign, regardless of the pre-test ROM. High suspicion ultrasound patterns may be associated with a >70% ROM and are found in the minority of nodules with indeterminate cytology (58,59,61,62,63,64). In nodules with such a high pre-test ROM, the NPV of Afirma is expected to be <70%, so it may not be useful to avoid surgery in such cases. If an Afirma GEC benign result is obtained in such a case, surgical hemithyroidectomy might be appropriate. Alternatively, an Afirma GEC suspicious result would be expected to further increase the ROM. Bethesda III/IV nodule with high suspicious ultrasound pattern is expected to have a ROM similar to the average Bethesda V (suspicious for malignancy) nodule. The 2015 ATA guideline indicates that patients with Bethesda V cytology should be treated similar to a malignant (Bethesda VI) nodule. Alternatively, nodules with very low, low, or intermediate ultrasound suspicion are associated with a malignancy risk of 20% or less. These ultrasound findings are expected in the vast majority of cytologically indeterminate nodules. In these nodules, the Afirma GEC would be expected to have an NPV of 96% or higher, and clinical observation in lieu of surgery may be appropriate in the majority of such patients. Those with Afirma GEC suspicious results may be considered for hemithyroidectomy based on their expected <40% ROM.

## FOLLOW-UP OF AFIRMA GENE EXPRESSION CLASSIFIER BENIGN PATIENTS

The 2015 ATA guidelines do not provide recommendations on the follow-up of cytologically indeterminate nodules that are Afirma GEC benign (13). Angell et al. (31) found that Afirma GEC benign nodules showed similar growth as cytopathology-benign cases, with malignancy found in only 1 Afirma GEC benign patient. The authors concluded that follow-up of Afirma GEC benign patients should be similar to that of cytology benign patients. The ATA guideline provides extensive detail and recommendations regarding the timing for follow-up for nodules with benign cytology that ranged from less than 12 months for those with high suspicion ultrasound patterns to potentially no follow-up for those with very low suspicion patterns (13). High suspicion sonographic pattern was recognized as a significantly better predictor of malignancy than nodule growth alone. Routine repeat FNAB was recommended only among cytologically benign nodules with high suspicion ultrasound patterns. For nodules with low or intermediate suspicion ultrasound patterns, only those that demonstrated growth or new suspicious sonographic features met criteria for repeat FNAB. The role of ultrasound follow-up for nodules with very low suspicion ultrasound patterns was less certain. For nodules found to be stable during follow-up the value of additional imaging was reported as low. The guideline suggested a diminishing frequency of additional ultrasound examinations for stable and asymptomatic nodules.

## COST-EFFECTIVENESS

An independent cost-effectiveness study found no difference in the number of missed cancers between paradigms with and without the Afirma GEC in a Markov model employing 10,000 Monte Carlo simulations of the expected range of probabilities for different potential outcomes (65). However, they did find that the Afirma paradigm reduced direct healthcare costs by $4,953 per five year episode of care, allowing $1,453 in direct savings using the then current Medicare reimbursement rates for surgery and the Afirma test, while modestly improving quality of life by 0.07 quality-adjusted life-year (QALY) (65). One criticism of this study has been the assumed test specificity of 75%, compared to the specificities of Afirma of 52% in Alexander et al. (4), as opposed to the specificity of 76% (95% CI 50-92%) in Chudova et al. (25). Still, cost savings/QALY was demonstrated in univariate analysis for specificity at the lowest value tested (60%) with cost savings and cost-effectiveness appearing likely at even lower specificities.

Lee et al. (66) modeled cost-effectiveness of the Afirma GEC and a 7-gene panel alone, and in combination, for Bethesda III nodules in the US and Canadian healthcare setting. In the US, the most cost-effective strategy was the Afirma GEC followed by the 7-gene panel in GEC suspicious cases, while in Canada management without molecular testing was most cost-effective. Wu et al. (48) compared routine Afirma GEC testing to conventional management in a decision tree model and found routine Afirma GEC testing more effective and most costly with an incremental cost-effectiveness ratio of $119,700/QALY, and found greater cost-effectiveness when either the prevalence of malignancy or the cost of the test were lowered. In Monte Carlo simulations, conventional management was the preferred strategy just over half the time. Base-case limitations of the both studies included that all Afirma GEC suspicious cases were directed to diagnostic hemithyroidectomy, and when malignant all cases then underwent completion thyroidectomy and added this significant cost. In practice, some patients may have elected total thyroidectomy and therefore avoided the added cost of completion thyroidectomy. In the model of Wu et al. (48), if more than just 3.1% of patients elected a total thyroidectomy instead of lobectomy in the absence of Afirma GEC testing then routine GEC testing became cost-effective. In a series of 165 Bethesda III/IV nodules operated without Afirma GEC testing, we reported that the use of total thyroidectomy was as low as 39% for Bethesda III nodules in academic centers to as high as 60% in Bethesda IV nodules in community practice settings [(67) supplemental data]. These data support the cost-effectiveness of the Afirma GEC as it can replace not only hemithyroidectomy, but can also significantly replace usage of the even more expensive total thyroidectomy with clinical observation. In addition, the mandated second (completion) surgery among malignant cases in the Lee (66) and Wu (48) models is not consistent with ATA and NCCN guidelines which suggest that thyroid lobectomy may be adequate treatment for most of these patients (13,60). Further, Lee et al. (66) added substantial penalties for delayed diagnosis when Afirma GEC benign patients were found to have cancer, including penalties for increased risk of cancer recurrence and death. These added costs are not consistent with the excellent outcome of known papillary thyroid cancer confined to the thyroid despite delayed treatment (68), or the excellent outcome of the few Afirma GEC false negative cases reported in the literature (31). Finally, the Lee et al. (66) model included significant costs for a yearly follow-up ultrasound examination of unoperated nodules, whereas the ATA guideline advocates for diminishing frequency of ultrasound follow-up over time (13).

Additional limitations of the 3 cost-effectiveness studies described above include that none consider indirect costs due to time lost from work and impacted responsibilities of daily living as a result of surgery and its recovery. Neither Lee et al. (66) nor Wu et al. (48) include costs for potential perioperative death, occurring in up to three in 1000 patients (8,69,70,71,72,73,74). The study methodologies may underestimate the impact of complications on the patient, including voice outcomes (75,76) and hypoparathyroidism (77). Additionally, all of the studies measure quality-adjusted life expectancies by multiplying the time spent in the health state by the utilities assigned to those states. The base-case utilities assigned to uncomplicated surgery are quite high and leave little room to improve quality of life by avoiding unnecessary diagnostic surgery. It does not seem correct that quality of life is diminished from surgery only when a complication occurs. Li et al. (65) assigned a higher base-case utility to an uncomplicated hemithyroidectomy than to observation, and the lower limit of the estimated utility range for observation was lower than the lower utility range of total thyroidectomy, suggesting that quality of life from observation could be worse than quality of life from an uncomplicated total thyroidectomy. These utility estimates (and those for complications) were derived from the opinions of people who have not undergone these procedures or experienced these complications. It seems likely that the value of avoiding diagnostic surgery may be greatly under-appreciated by those who have not actually experienced the event than those that have, a finding shown to be true for hypoparathyroidism (77). Future research is needed to better quantify relevant utility values so that changes in quality of life resulting from changes in patient care can be better measured.

## MALIGNANCY CLASSIFIERS

While the current greatest value of molecular diagnostics among cytologically indeterminate nodules is to identify nodules that do not require surgery (a rule-out test), there is value to a test that can identify malignancy (a rule-in test) only when it alters clinical care to the benefit of the patient. Clinical care can be altered by enhancing the rationale for surgery, and more directly by altering the extent of surgical care (11). The Afirma Malignancy Classifiers include a *BRAF^V600E^* point mutation classifier, and a cassette for MTC. Additional cassettes automatically run with every Afirma GEC test screen for parathyroid tissue (benign and malignancy) (78), and metastases to the thyroid from malignant melanoma, breast, and renal cell carcinomas.

MTC is frequently a cytological challenge to diagnose, and the field has had attention recently drawn to the low sensitivity of FNAB for the specific diagnosis of MTC (79,80). MTC cases are found among all 6 Bethesda cytological categories. When MTC is not recognized pre-operatively then delayed diagnosis may result (79), and those that undergo surgery may not be pre-operatively evaluated for MEN2 associated hyperparathyroidism, or concomitant pheochromocytoma (81). Surgery on a patient with an unrecognized pheochromocytoma may result in death. MTC that is not specifically recognized pre-operatively as MTC is unlikely to undergo the optimal initial surgery, typically considered to be a total thyroidectomy and central neck dissection at a minimum (81). In a recently study, only 18.7% of MTC patients underwent surgery for an accurate diagnosis (79). The Afirma MTC classifier has been evaluated in patients and tissue, and has exceptionally high sensitivity (96%), specificity (>99%), PPV (98%), and NPV (>99%) (82,83). With more than 40,000 Afirma GEC tests performed, Veracyte is aware of only one MTC case that was GEC suspicious, but not identified by the classifier as MTC, and 1 false positive case, an intra-parathyroidal, intra-thyroidal paraganglioma (82,84). The MTC classifier is routinely run and reported with every GEC test globally. In the US, the MTC classifier may be obtained without the Afirma GEC on Bethesda V or VI nodules.

Point mutations in *BRAF* are by far the most common genomic abnormality associated with papillary thyroid carcinomas, and nearly all are *BRAF^V600E^* mutations (85). The Afirma *BRAF^V600E^* classifier is based on the mRNA molecular signature of 128 genes (86). Compared to a sensitive quantitative PCR assay, high positive and negative percent agreement was demonstrated (PPA 90.4% and NPA 99.0%). Establishing appropriate cut-off points to separate positive from negative tests is critical to avoid false positive results than can drive inappropriate treatment (87). When cut-offs are correctly established, *BRAF^V600E^* mutation is uncommon among Bethesda III and IV nodules, while it is more common among Bethesda V and VI nodules (67,86). Unlike RAS mutations (14,15,16,17,18,19,20,21), *BRAF^V600E^* mutations are almost exclusively found in malignant nodules (e.g. PPV ~100%). In a consecutive cohort of 7,066 de-identified FNABs, 3,187 samples were benign by Afirma GEC, of which none were Afirma BRAF positive (88). Thus, testing only Afirma GEC suspicious samples increases the rate of positive tests and decreases healthcare costs. The Afirma *BRAF^V600E^* classifier is accurate among samples that constitute up to 60% blood. Interestingly, a double-mutant that resulted in the V600E amino acid change but was negative by qPCR but was identified by the Afirma BRAF classifier. The non-diagnostic rates were lower (7.6%) for Afirma BRAF than for qPCR (24.5%), a further advantage of using RNA in FNAB small sample biopsies. In the US, the *BRAF^V600E^* classifier is an option on Afirma GEC suspicious nodules, and Bethesda V or VI nodules without Afirma GEC.

## CONCLUSION

Cytologically indeterminate nodules have historically been referred for surgery given that their ROM was above the typical threshold of ~5% for physicians to consider clinical observation in lieu of diagnostic surgery. Molecular diagnostic testing of these nodules has rapidly become accepted. Current guidelines include that molecular testing may be used among Bethesda III/IV nodules to add additional information about the nodule’s ROM. The 2015 ATA guideline reviews the molecular testing landscape, and voices caution over tests supported only by single center and unblinded validation data, and those with no published clinical utility data to demonstrate a change in clinical care and patient benefit as a result of the test. The Afirma GEC is the only molecular test supported by multicenter, prospective, and blinded validation data, and the only test supported by published clinical utility data demonstrating a dramatic reduction in diagnostic surgery for patients with benign Afirma GEC results. Nearly 1 out of every 2 Afirma GEC tests performed yields a molecularly benign result, and >80% of patients with a benign GEC result remain unoperated 3 years after the biopsy in real-world experience. Reducing unnecessary diagnostic surgery improves patient safety, reduces healthcare costs, and improves patient quality of life.

## References

[1] Cooper DS, Doherty GM, Haugen BR, Kloos RT, Lee SL, Mandel SJ, Mazzaferri EL, McIver B, Pacini F, Schlumberger M, Sherman SI, Steward DL, Tuttle RM, American Thyroid Association (ATA) Guidelines Taskforce on Thyroid Nodules and Differentiated Thyroid Cancer (2009). Revised American Thyroid Association management guidelines for patients with thyroid nodules and differentiated thyroid cancer. Thyroid.

[2] Hegedüs L (2004). Clinical practice. The thyroid nodule. N Engl J Med.

[3] Melillo RM, Santoro M (2012). Molecular biomarkers in thyroid FNA samples. J Clin Endocrinol Metab.

[4] Alexander EK, Kennedy GC, Baloch ZW, Cibas ES, Chudova D, Diggans J, Friedman L, Kloos RT, LiVolsi VA, Mandel SJ, Raab SS, Rosai J, Steward DL, Walsh PS, Wilde JI, Zeiger MA, Lanman RB, Haugen BR (2012). Preoperative diagnosis of benign thyroid nodules with indeterminate cytology. N Engl J Med.

[5] Wang CC, Friedman L, Kennedy GC, Wang H, Kebebew E, Steward DL, Zeiger MA, Westra WH, Wang Y, Khanafshar E, Fellegara G, Rosai J, Livolsi V, Lanman RB (2011). A large multicenter correlation study of thyroid nodule cytopathology and histopathology. Thyroid.

[6] Bryson PC, Shores CG, Hart C, Thorne L, Patel MR, Richey L, Farag A, Zanation AM (2008). Immunohistochemical distinction of follicular thyroid adenomas and follicular carcinomas. Arch Otolaryngol Head Neck Surg.

[7] Cibas ES, Ali SZ (2009). The Bethesda system for reporting thyroid cytopathology. Thyroid.

[8] Weiss A, Parina RP, Tang JA, Brumund KT, Chang DC, Bouvet M (2015). Outcomes of thyroidectomy from a large California state database. Am J Surg.

[9] Zhu QL, Faquin WC, Samir AE (2015). Relationship between sonographic characteristics and afirma gene expression classifier results in thyroid nodules with indeterminate fine-needle aspiration cytopathology. AJR Am J Roentgenol.

[10] Kuru B, Atmaca A, Tarim IA, Kefeli M, Topgul K, Yoruker S, Elmali M, Danaci M (2015). Risk factors associated with malignancy and with triage to surgery in thyroid nodules classified as Bethesda category III (AUS/FLUS). Eur J Surg Oncol.

[11] Nishino M (2016). Molecular cytopathology for thyroid nodules: A review of methodology and test performance. Cancer Cytopathol.

[12] Xing M, Haugen BR, Schlumberger M (2013). Progress in molecular-based management of differentiated thyroid cancer. Lancet.

[13] Haugen BR, Alexander EK, Bible KC, Doherty GM, Mandel SJ, Nikiforov YE, Pacini F, Randolph GW, Sawka AM, Schlumberger M, Schuff KG, Sherman SI, Sosa JA, Steward DL, Tuttle RM, Wartofsky L (2016). 2015 American Thyroid Association Management Guidelines for Adult Patients with Thyroid Nodules and Differentiated Thyroid Cancer: The American Thyroid Association Guidelines Task Force on Thyroid Nodules and Differentiated Thyroid Cancer. Thyroid.

[14] Cantara S, Capezzone M, Marchisotta S, Capuano S, Busonero G, Toti P, Santo A, Caruso G, Carli AF, Brilli L, Montanaro A, Pacini F (2010). Impact of proto-oncogene mutation detection in cytological specimens from thyroid nodules improves the diagnostic accuracy of cytology. J Clin Endocrinol Metab.

[15] Eszlinger M, Krogdahl A, Münz S, Rehfeld C, Precht Jensen EM, Ferraz C, Bösenberg E, Drieschner N, Scholz M, Hegedüs L, Paschke R (2014). Impact of molecular screening for point mutations and rearrangements in routine air-dried fine-needle aspiration samples of thyroid nodules. Thyroid.

[16] Eszlinger M, Piana S, Moll A, Bösenberg E, Bisagni A, Ciarrocchi A, Ragazzi M, Paschke R (2015). Molecular testing of thyroid fine-needle aspirations improves presurgical diagnosis and supports the histologic identification of minimally invasive follicular thyroid carcinomas. Thyroid.

[17] Rossi M, Buratto M, Tagliati F, Rossi R, Lupo S, Trasforini G, Lanza G, Franceschetti P, Bruni S, Degli Uberti E, Zatelli MC (2015). Relevance of BRAF(V600E) mutation testing versus RAS point mutations and RET/PTC rearrangements evaluation in the diagnosis of thyroid cancer. Thyroid.

[18] Moses W, Weng J, Sansano I, Peng M, Khanafshar E, Ljung BM, Duh QY, Clark OH, Kebebew E (2010). Molecular testing for somatic mutations improves the accuracy of thyroid fine-needle aspiration biopsy. World J Surg.

[19] Krane JF, Cibas ES, Alexander EK, Paschke R, Eszlinger M (2015). Molecular analysis of residual ThinPrep material from thyroid FNAs increases diagnostic sensitivity. Cancer Cytopathol..

[20] Medici M, Kwong N, Angell TE, Marqusee E, Kim MI, Frates MC, Benson CB, Cibas ES, Barletta JA, Krane JF, Ruan DT, Cho NL, Gawande AA, Moore FD, Alexander EK (2015). The variable phenotype and low-risk nature of RAS-positive thyroid nodules. BMC Med.

[21] Gill MS, Nayan S, Kocovski L, Cutz JC, Archibald SD, Jackson BS, Young JE, Gupta MK (2015). Local molecular analysis of indeterminate thyroid nodules. J Otolaryngol Head Neck Surg.

[22] Kloos R, Walsh P, Pagan M, Matsuzaki H, Huang J, Travers K, Tom E, Wong M, Mathison J, Kim S, Kennedy G (2015). Detection of increasing numbers of point mutations and fusions in benign thyroid nodules using next-generation sequencing dramatically reduces mutation marker panel specificity (abstract). Endocrine Practice.

[23] Pagan M, Kloos RT, Lin CF, Travers KJ, Matsuzaki H, Tom EY, Kim SY, Wong MG, Stewart AC, Huang J, Walsh PS, Monroe RJ, Kennedy GC (2016). The Diagnostic application of RNA sequencing in patients with thyroid cancer: an analysis of 851 variants and 133 fusions in 524 genes. BMC Bioinformatics.

[24] Grody WW, Thompson BH, Hudgins L (2013). Whole-exome/genome sequencing and genomics. Pediatrics.

[25] Chudova D, Wilde JI, Wang ET, Wang H, Rabbee N, Egidio CM, Reynolds J, Tom E, Pagan M, Rigl CT, Friedman L, Wang CC, Lanman RB, Zeiger M, Kebebew E, Rosai J, Fellegara G, LiVolsi VA, Kennedy GC (2010). Molecular classification of thyroid nodules using high-dimensionality genomic data. J Clin Endocrinol Metab.

[26] Shrestha M, Crothers BA, Burch HB (2012). The impact of thyroid nodule size on the risk of malignancy and accuracy of fine-needle aspiration: a 10-year study from a single institution. Thyroid.

[27] Lewis CM, Chang KP, Pitman M, Faquin WC, Randolph GW (2009). Thyroid fine-needle aspiration biopsy: variability in reporting. Thyroid.

[28] Borget I, Vielh P, Leboulleux S, Allyn M, Iacobelli S, Schlumberger M, Pouvourville G (2008). Assessment of the cost of fine-needle aspiration cytology as a diagnostic tool in patients with thyroid nodules. Am J Clin Pathol.

[29] Renshaw A (2010). An estimate of risk of malignancy for a benign diagnosis in thyroid fine-needle aspirates. Cancer Cytopathol.

[30] Marchevsky AM, Walts AE, Bose S, Gupta R, Fan X, Frishberg D, Scharre K, Zhai J (2010). Evidence-based evaluation of the risks of malignancy predicted by thyroid fine-needle aspiration biopsies. Diagn Cytopathol.

[31] Angell TE, Frates MC, Medici M, Liu X, Kwong N, Cibas ES, Kim MI, Marqusee E (2015). Afirma benign thyroid nodules show similar growth to cytologically benign nodules during follow-up. J Clin Endocrinol Metab.

[32] (December 10, 2015). NCCN Clinical Practice Guidelines in Oncology.

[33] Beaudenon-Huibregtse S, Alexander EK, Guttler RB, Hershman JM, Babu V, Blevins TC, Moore P, Andruss B, Labourier E (2014). Centralized molecular testing for oncogenic gene mutations complements the local cytopathologic diagnosis of thyroid nodules. Thyroid.

[34] Nikiforov YE, Ohori NP, Hodak SP, Carty SE, LeBeau SO, Ferris RL, Yip L, Seethala RR, Tublin ME, Stang MT, Coyne C, Johnson JT, Stewart AF, Nikiforova MN (2011). Impact of mutational testing on the diagnosis and management of patients with cytologically indeterminate thyroid nodules: a prospective analysis of 1056 FNA samples. J Clin Endocrinol Metab.

[35] Kumar G, Song-Yang J, Timmaraju VA, Taylor S, Mireskandari A, Finkelstein S, Hosono S (2015). Thyroid mirna classifier (ThyraMir) complements mutation detection (ThyGenX) NGS tests for improved molecular diagnosis of indeterminate thyroid nodule needle aspirates. Thyroid..

[36] Nikiforov YE, Carty SE, Chiosea SI, Coyne C, Duvvuri U, Ferris RL, Gooding WE, LeBeau SO, Ohori NP, Seethala RR, Tublin ME, Yip L, Nikiforova MN (2015). Impact of the multi-gene thyroseq next-generation sequencing assay on cancer diagnosis in thyroid nodules with atypia of undetermined significance/follicular lesion of undetermined significance cytology. Thyroid.

[37] Nikiforov YE, Carty SE, Chiosea SI, Coyne C, Duvvuri U, Ferris RL, Gooding WE, Hodak SP, LeBeau SO, Ohori NP, Seethala RR, Tublin ME, Yip L, Nikiforova MN (2014). Highly accurate diagnosis of cancer in thyroid nodules with follicular neoplasm/suspicious for a follicular neoplasm cytology by ThyroSeq v2 next-generation sequencing assay. Cancer.

[38] Bar D, Yania GL, Goren Y, Shtabsky A, Zubkov A, Morgenstern S, Strenov Y, Feinmesser M, Kravtsov V, Leon M, Granstrem O, Vorobyov S, Hajduch M, VandenBussche C, Ashkenazi K, Sanden M, Mitchell H, Noller M, Dromi N, Tabak S, Kadosh E, Meiri E (2015). A first-of-its-kind, microRNA-based diagnostic assay for accurate thyroid nodule classification. Thyroid.

[39] Teutsch SM, Bradley LA, Palomaki GE, Haddow JE, Piper M, Calonge N, Dotson WD, Douglas MP, Berg AO, EGAPP Working Group (2009). The evaluation of genomic applications in practice and prevention (EGAPP) initiative: methods of the EGAPP Working Group. Genet Med.

[40] Duick DS, Klopper JP, Diggans JC, Friedman L, Kennedy GC, Lanman RB, McIver B (2012). The impact of benign gene expression classifier test results on the endocrinologist-patient decision to operate on patients with thyroid nodules with indeterminate fine-needle aspiration cytopathology. Thyroid.

[41] Alexander EK, Schorr M, Klopper J, Kim C, Sipos J, Nabhan F, Parker C, Steward DL, Mandel SJ, Haugen BR (2014). Multicenter clinical experience with the Afirma gene expression classifier. J Clin Endocrinol Metab.

[42] Harrell RM, Bimston DN (2014). Surgical utility of Afirma: effects of high cancer prevalence and oncocytic cell types in patients with indeterminate thyroid cytology. Endocr Pract.

[43] Sipos JA, Blevins TC, Shea HC, Duick DS, Lakhian SK, Michael BE, Thomas MJ, Sosa JA (2016). Long-term non-operative rate of thyroid nodules with benign results on the afirma gene expression classifier.

[44] Sullivan PS, Hirschowitz SL, Fung PC, Apple SK (2014). The impact of atypia/follicular lesion of undetermined significance and repeat fine-needle aspiration: 5 years before and after implementation of the Bethesda System. Cancer Cytopathol.

[45] Witt RL (2016). Outcome of thyroid gene expression classifier testing in clinical practice. Laryngoscope.

[46] Marti JL, Avadhani V, Donatelli LA, Niyogi S, Wang B, Wong RJ, Shaha AR, Ghossein RA, Lin O, Morris LG, Ho AS (2015). Wide Inter-institutional variation in performance of a molecular classifier for indeterminate thyroid nodules. Ann Surg Oncol.

[47] Lastra RR, Pramick MR, Crammer CJ, LiVolsi VA, Baloch ZW (2014). Implications of a suspicious afirma test result in thyroid fine-needle aspiration cytology: an institutional experience. Cancer Cytopathol.

[48] Wu JX, Lam R, Levin M, Rao J, Sullivan PS, Yeh MW (2016). Effect of malignancy rates on cost-effectiveness of routine gene expression classifier testing for indeterminate thyroid nodules. Surgery.

[49] Celik B, Whetsell CR, Nassar A (2015). Afirma GEC and thyroid lesions: An institutional experience. Diagn Cytopathol.

[50] Yang SE, Sullivan PS, Zhang J, Govind R, Levin MR, Rao JY, Moatamed NA (2016). Has Afirma gene expression classifier testing refined the indeterminate thyroid category in cytology?. Cancer Cytopathol.

[51] McIver B, Castro MR, Morris JC, Bernet V, Smallridge R, Henry M, Kosok L, Reddi H (2014). An independent study of a gene expression classifier (Afirma) in the evaluation of cytologically indeterminate thyroid nodules. J Clin Endocrinol Metab.

[52] Cibas ES, Baloch ZW, Fellegara G, LiVolsi VA, Raab SS, Rosai J, Diggans J, Friedman L, Kennedy GC, Kloos RT, Lanman RB, Mandel SJ, Sindy N, Steward DL, Zeiger MA, Haugen BR, Alexander EK (2013). A prospective assessment defining the limitations of thyroid nodule pathologic evaluation. Ann Intern Med.

[53] Steward DL, Kloos RT (2014). Clinical diagnostic gene expression thyroid testing. Otolaryngol Clin North Am.

[54] Noureldine SI, Olson MT, Agrawal N, Prescott JD, Zeiger MA, Tufano RP (2015). Effect of gene expression classifier molecular testing on the surgical decision-making process for patients with thyroid nodules. JAMA Otolaryngol Head Neck Surg.

[55] Keutgen XM, Filicori F, Crowley MJ, Wang Y, Scognamiglio T, Hoda R, Buitrago D, Cooper D, Zeiger MA, Zarnegar R, Elemento O, Fahey TJ (2012). A panel of four miRNAs accurately differentiates malignant from benign indeterminate thyroid lesions on fine needle aspiration. Clin Cancer Res.

[56] Brauner E, Holmes BJ, Krane JF, Nishino M, Zurakowski D, Hennessey JV, Faquin WC, Parangi S (2015). Performance of the afirma gene expression classifier in hurthle cell thyroid nodules differs from other indeterminate thyroid nodules. Thyroid.

[57] VanderLaan PA, Marqusee E, Krane JF (2011). Clinical outcome for atypia of undetermined significance in thyroid fine-needle aspirations: should repeated fna be the preferred initial approach?. Am J Clin Pathol.

[58] Rosario PW (2014). Thyroid nodules with atypia or follicular lesions of undetermined significance (Bethesda Category III): importance of ultrasonography and cytological subcategory. Thyroid.

[59] Maia FF, Matos PS, Pavin EJ, Vassallo J, Zantut-Wittmann DE (2011). Value of ultrasound and cytological classification system to predict the malignancy of thyroid nodules with indeterminate cytology. Endocr Pathol.

[60] Tuttle RM, Haddad RI, Ball DW, Byrd D, Dickson P, Duh QY, Ehya H, Haymart M, Hoh C, Hunt JP, Iagaru A, Kandeel F, Kopp P, Lamonica DM, Lydiatt WM, McCaffrey J, Moley JF, Parks L, Raeburn CD, Ridge JA, Ringel MD, Scheri RP, Shah JP, Sherman SI, Sturgeon C, Waguespack SG, Wang TN, Wirth LJ, Hoffmann KG, Hughes M (2014). Thyroid carcinoma, version 2.2014. J Natl Compr Canc Netw.

[61] Rosario PW, Salles DS, Bessa B, Purisch S (2010). Contribution of scintigraphy and ultrasonography to the prediction of malignancy in thyroid nodules with indeterminate cytology. Arq Bras Endocrinol Metabol.

[62] Kim DW, Lee EJ, Jung SJ, Ryu JH, Kim YM (2011). Role of sonographic diagnosis in managing Bethesda class III nodules. AJNR Am J Neuroradiol.

[63] Chng CL, Kurzawinski TR, Beale T (2015). Value of sonographic features in predicting malignancy in thyroid nodules diagnosed as follicular neoplasm on cytology. Clin Endocrinol (Oxf).

[64] Russ G, Royer B, Bigorgne C, Rouxel A, Bienvenu-Perrard M, Leenhardt L (2013). Prospective evaluation of thyroid imaging reporting and data system on 4550 nodules with and without elastography. Eur J Endocrinol.

[65] Li H, Robinson KA, Anton B, Saldanha IJ, Ladenson PW (2011). Cost-effectiveness of a novel molecular test for cytologically indeterminate thyroid nodules. J Clin Endocrinol Metab.

[66] Lee L, How J, Tabah RJ, Mitmaker EJ (2014). Cost-effectiveness of molecular testing for thyroid nodules with atypia of undetermined significance cytology. J Clin Endocrinol Metab.

[67] Kloos RT, Reynolds JD, Walsh PS, Wilde JI, Tom EY, Pagan M, Barbacioru C, Chudova DI, Wong M, Friedman L, LiVolsi VA, Rosai J, Lanman RB, Kennedy GC (2013). Does addition of BRAF V600E mutation testing modify sensitivity or specificity of the afirma gene expression classifier in cytologically indeterminate thyroid nodules?. J Clin Endocrinol Metab.

[68] Davies L, Welch HG (2010). Thyroid cancer survival in the United States: observational data from 1973 to 2005. Arch Otolaryngol Head Neck Surg.

[69] Shrime MG, Goldstein DP, Seaberg RM, Sawka AM, Rotstein L, Freeman JL, Gullane PJ (2007). Cost-effective management of low-risk papillary thyroid carcinoma. Arch Otolaryngol Head Neck Surg.

[70] Hundahl SA, Cady B, Cunningham MP, Mazzaferri E, McKee RF, Rosai J, Shah JP, Fremgen AM, Stewart AK, Hölzer S (2000). Initial results from a prospective cohort study of 5583 cases of thyroid carcinoma treated in the united states during 1996. U.S. and German Thyroid Cancer Study Group. An American College of Surgeons Commission on Cancer Patient Care Evaluation study. Cancer.

[71] Bergenfelz A, Jansson S, Kristoffersson A, Martensson H, Reihner E, Wallin G, Lausen I (2008). Complications to thyroid surgery: results as reported in a database from a multicenter audit comprising 3,660 patients. Langenbecks Arch Surg.

[72] Sosa JA, Bowman HM, Tielsch JM, Powe NR, Gordon TA, Udelsman R (1998). The importance of surgeon experience for clinical and economic outcomes from thyroidectomy. Ann Surg.

[73] Hauch A, Al-Qurayshi Z, Randolph G, Kandil E (2014). Total thyroidectomy is associated with increased risk of complications for low- and high-volume surgeons. Ann Surg Oncol.

[74] Weiss A, Lee KC, Brumund KT, Chang DC, Bouvet M (2014). Risk factors for hematoma after thyroidectomy: results from the nationwide inpatient sample. Surgery.

[75] Vicente DA, Solomon NP, Avital I, Henry LR, Howard RS, Helou LB, Coppit GL, Shriver CD, Buckenmaier CC, Libutti SK, Shaha AR, Stojadinovic A (2014). Voice outcomes after total thyroidectomy, partial thyroidectomy, or non-neck surgery using a prospective multifactorial assessment. J Am Coll Surg.

[76] Kuhn MA, Bloom G, Myssiorek D (2013). Patient perspectives on dysphonia after thyroidectomy for thyroid cancer. J Voice.

[77] Cho NL, Moalem J, Chen L, Lubitz CC, Moore FD, Ruan DT (2014). Surgeons and patients disagree on the potential consequences from hypoparathyroidism. Endocr Pract.

[78] Kloos R, Harrell RM, Kennedy GC, Monroe RJ, Traweek ST, Lanman RB (2014). Preoperative identification of parathyroid tissue by an mRNA classifier on prospectively collected thyroid nodule fine-needle aspiration biopsies.

[79] Ha EJ, Baek JH, Na DG, Kim JH, Kim JK, Min HS, Song DE, Lee KE, Shong YK (2015). The Role of core needle biopsy and Its impact on surgical management in patients with medullary thyroid cancer: clinical experience at 3 medical institutions. AJNR Am J Neuroradiol.

[80] Trimboli P, Treglia G, Guidobaldi L, Romanelli F, Nigri G, Valabrega S, Sadeghi R, Crescenzi A, Faquin WC, Bongiovanni M, Giovanella L (2015). Detection rate of FNA cytology in medullary thyroid carcinoma: a meta-analysis. Clin Endocrinol (Oxf).

[81] Wells SA, Asa SL, Dralle H, Elisei R, Evans DB, Gagel RF, Lee N, Machens A, Moley JF, Pacini F, Raue F, Frank-Raue K, Robinson B, Rosenthal MS, Santoro M, Schlumberger M, Shah M, Waguespack SG, American Thyroid Association Guidelines Task Force on Medullary Thyroid Carcinoma (2015). Revised American Thyroid Association guidelines for the management of medullary thyroid carcinoma. Thyroid.

[82] Kloos RT, Kennedy G, Monroe R, Traweek ST, Lanman R (2014). Validation of a molecular classifier for preoperative identification of medullary thyroid cancer in thyroid nodule fine-needle aspiration biopsies.

[83] Pankratz D, Hu Z, Kim SY, Monroe R, Wong M, Diggans J, Traweek T, Kumm J, Lanman R, Kloos R, Walsh S, Kennedy K (2014). Analytical validation of a gene expression classifier for medullary thyroid carcinoma.

[84] Shvets L, Lubitz S (2014). A unique case of intrathyroidal intraparathyroidal paraganglioma.

[85] Cancer Genome Atlas Research Network (2014). Integrated genomic characterization of papillary thyroid carcinoma. Cell.

[86] Diggans J, Kim SY, Hu Z, Pankratz D, Wong M, Reynolds J, Tom E, Pagan M, Monroe R, Rosai J, Livolsi VA, Lanman RB, Kloos RT, Walsh PS, Kennedy GC (2015). Machine learning from concept to clinic: reliable detection of BRAF V600e DNA mutations in thyroid nodules using high-dimensional RNA expression data. Pac Symp Biocomput.

[87] DiLorenzo MM, Miller JL, Tuluc M, Wang ZX, Savarese VW, Pribitkin EA (2014). False-positive FNA due to highly sensitive BRAF assay. Endocr Pract.

[88] Walsh P, Diggans J, Hu Z, Kim SY, Pankratz D, Tom E, Wilde J, Reynolds J, Velichko S, Wong M, Mathison J, Kloos R, Lanman R, Kennedy G (2014). Pre-operative identification of BRAF V600E mutational status in thyroid nodules using a novel gene expression classifier.

